# Role of the protein in the DNA sequence specificity of the cleavage site stabilized by the camptothecin topoisomerase IB inhibitor: a metadynamics study

**DOI:** 10.1093/nar/gkt790

**Published:** 2013-09-03

**Authors:** Andrea Coletta, Alessandro Desideri

**Affiliations:** Dipartimento di Biologia, Universitá degli Studi di Roma ‘Tor Vergata’, Via della Ricerca Scientifica, 00133 Roma, Italy

## Abstract

Camptothecin (CPT) is a topoisomerase IB (TopIB) selective inhibitor whose derivatives are currently used in cancer therapy. TopIB cleaves DNA at any sequence, but in the presence of CPT the only stabilized protein–DNA covalent complex is the one having a thymine in position −1 with respect to the cleavage site. A metadynamics simulation of two TopIB–DNA–CPT ternary complexes differing for the presence of a thymine or a cytosine in position −1 indicates the occurrence of two different drug’s unbinding pathways. The free-energy difference between the bound state and the transition state is large when a thymine is present in position −1 and is strongly reduced in presence of a cytosine, in line with the different drug stabilization properties of the two systems. Such a difference is strictly related to the changes in the hydrogen bond network between the protein, the DNA and the drug in the two systems, indicating a direct role of the protein in determining the specificity of the cleavage site sequence stabilized by the CPT. Calculations carried out in presence of one compound of the indenoisoquinoline family (NSC314622) indicate a comparable energy difference between the bound and the transition state independently of the presence of a thymine or a cytosine in position −1, in line with the experimental results.

## INTRODUCTION

Topoisomerase IB (TopIB) is an ubiquitous enzyme belonging to the class of DNA topoisomerases, a family of protein involved in the regulation of DNA topology that has a crucial role in removing DNA supercoiling occurring during replication and transcription (1,2). TopIB catalytic cycle is conventionally divided in five steps: (i) DNA binding, (ii) DNA cleavage, (iii) DNA relaxation, (iv) DNA religation and (v) DNA unbinding. In detail, TopIB cleaves one strand of a double-stranded DNA through a nucleophilic attack operated by the Tyr723 residue resulting in a DNA-3′-phosho-tyrosine covalent bond. The cleavage permits the rotation of DNA downstream of the nicked site around the intact strand, through the so-called ‘Controlled Rotation’ mechanism (2). After the energy equilibrium of supercoiled DNA is reached through modification of the DNA linking number, a second nucleophilic attack by the O5′ DNA end restores an intact double strand and the enzyme is released.

TopIB is of considerable medical interest, being an effective target of many natural and nonnatural compounds (3), and among these, camptothecin (CPT), a quinoline alkaloid first isolated in 1966 from *Camptotheca acuminata*, behaves as a selective uncompetitive inhibitor (4). CPT does not bind to DNA or TopIB alone but it selectively interacts with the TopIB–DNA covalent complex, stabilizing it, intercalating between the base pairs in position −1 and +1 with respect to the cleavage site, slowing down the DNA relaxation and religation. As a result, a fully active enzyme is required for CPT action (5). The stabilization of the TopIB–DNA complex leads, in highly proliferating cell, to DNA damage as a consequence of a clash with the DNA replication machinery, eventually triggering the apoptotic pathways in cancer cells.

Human TopIB does not have a consensus sequence for DNA binding and cleavage, but in presence of CPT the cleavage pattern of TopIB shows a preference for a guanine in position +1 and the requirement of a thymine in position −1 (6). Indenoisoquinolines (IQNs), a new class of TopIB inhibitors currently undergoing Phase I clinical trial, stabilize TopIB also when a cytosine is present in position −1 (7,8). Attempts in providing an explanation for the sequence preference of CPT stabilization at a preferred cleavage site have been made using Quantum Mechanics single point energy calculations (9–13). Umbrella sampling molecular dynamics (MD) simulation coupled with Molecular Mechanics Generalized Born Surface Area energy decomposition on simplified drug–DNA systems have shown that the apolar nonbonding interaction between CPT and DNA bases at the cleavage site plays a role in the stabilization of the complex (14).

However, the analysis of available crystallographic structures of TopIB ternary complexes (15–19) as well as long MD simulations (20,21) have shown that the stabilization of the inhibitors is also related to the formation of a hydrogen bond network between the protein, the DNA and the drug, indicating that the role of the protein must be taken into account to fully understand the mechanism of CPT sequence specificity. Metadynamics is a powerful simulative tool that permits to overcome some intrinsic limitations of MD simulations, introducing an adaptively biased potential energy that progressively permits the system under investigation to explore the free-energy surface (FES) as a function of previously determined collective variables (CVs) (22,23). The method has been tested in biologically relevant systems, and recently a well-tempered variant of metadynamics, ensuring a correct reconstruction of FES, has been developed (24). Metadynamics have been successfully used to study the unbinding process of small DNA intercalators and small protein inhibitors (25–27), but up to now it has never been applied to the study of the unbinding of a drug from a protein–DNA complex.

In this work we present two metadynamics simulations of the ternary TopIB–DNA–CPT complex, following the CPT unbinding in two different situations, namely when in position −1 of the cleavage site a thymine or a cytosine is present. The same two metadynamics simulations carried out on the Top1B–DNA–IQN ternary system indicate a comparable unbinding energy independently of the presence of a thymine or a cytosine in position −1, in line with what was experimentally found. The analysis permits to propose an explanation, at the atomistic level, of the strict preference of CPT to stabilize the cleaved site having a thymine in position −1 and shows the important interplay between the protein and DNA in stabilizing the drug.

## MATERIALS AND METHODS

### System preparation and MD protocol

The starting model for the TopIB–DNA–CPT ternary complex containing a thymine in position −1 has been obtained from the last frame of a 25-ns simulation of the TopIB–DNA–TPT ternary complex previously published (21). The TPT substituents on A-ring have been removed and the CPT point charges parametrization have been performed using the Restrained Electrostatic Potential procedure (28) as previously reported (29–31). On the cleavage site of this system, in position −1 and +1, an A:T and a C:G base pairs are present (the first base of each couple belonging to the intact strand). This configuration has been named ‘TG system’. A second system has been built mutating the base pair in position −1 from A:T to G:C leading to the ‘CG system’. The two systems have been equilibrated for 4.0 ns with a NPT (Isobaric-Isothermal ensemble) MD run. The sampling of canonical ensemble at 300K has been ensured by the coupling with an external bath using the velocity rescaling Berensen thermostat (32) with a time constant of 2.0 ps. The Parrinello-Rahman barosthat (33) has been used to maintain the pressure of the system to 1 bar with a time constant of 2.0 ps. All the bond lengths of the protein and DNA have been constrained to their equilibrium values using the Linear Constraints Solver algorithm (34), while water geometry has been constrained using the SETTLE algorithm (35). The equation of motion has been integrated using the leap-frog algorithm with a 2.0-fs time steps. The trajectory has been saved every 250 steps (0.5 ps).

For the simulation of IQN, the same coordinate of TopIB and DNA used to build the CPT systems have been used. The IQN (NSC314622) has been positioned in the binding site using as a reference the coordinate of the analog MJ-238 in PDB entry 1SC7 (17). The system has been thermalized as previously reported (20) and then subjected to a 4.0-ns equilibration before starting the metadynamics simulation.

MD have been carried out using Gromacs-4.5.5 (36).

### Metadynamics

The unbinding of CPT or IQN from the TG and CG equilibrated systems has been simulated by means of metadynamics (23) using Gromacs-4.5.5 with the PLUMEDv1.3 patch (37). Metadynamics is a simulative technique that enhances the sampling of high-energy regions of the FES by adding to the total energy of the system a biasing history dependent term, obtained by the summation of Gaussian hills laying on the subspace identified by a set of user-defined CVs (22). The Gaussian hills are added at a constant time interval in the position explored by the system in the space of CVs. The most visited regions of conformational space (low free energy) are slowly ‘filled’ and the biasing potential enhances the exploration of the less probable (high free energy) regions. The FES can be reconstructed at the end of simulation as the summation of the added Gaussian hills. The well-tempered variant of metadynamics is an adaptative-biased method that permits, through a progressive reduction of Gaussian hills height, a correct convergence of FES calculation (24).

In the simulation, two CVs have been used to describe the inhibitor unbinding:

1. The distance between the center of mass of the inhibitor and of the nucleobases of stacking nucleotides in position −1 and +1.
2. The number of contacts between the inhibitor and the nucleobases of nucleotides in position −1 and +1, counted using the PLUMED implemented CV coordination number (CN):

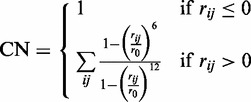

where 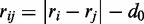, (*r_i_* and *r_j_* being the coordinates of the inhibitor and nucleobases atoms, respectively). The value of the parameter *d*_0_ has been set to 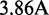, corresponding to the position of the first peak of the radial atom-pair distribution function of inhibitor and nucleobases atoms distances, calculated in the 4.0 ns equilibration MD. The *r*_0_ value has been set to 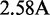, corresponding to the full width at half maximum of the same distribution.

The Gaussian hills have a height of 1.0 kJ/mole, a width of 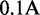 and 50 in the first and second CV dimension, respectively. During metadynamics the hills have been deposed every 250 frames (0.5 ps). The FES reconstruction has been carried out summing 27 644 (13.8 ns) and 33 211 (16.6 ns) Gaussian hills for the TG and the CG system, respectively, and has been saved on a 500 x 500 grid. The summation has been stopped when the inhibitor has reached a reference distance of 4.0 nm from the binding site, i.e. when the inhibitor is no longer contacting either the protein or the DNA.

The data here obtained cannot be related to the dissociation constant of the inhibitor because a calculation of *K*_d_ would require a full sampling of the unbound state. However, the free-energy difference between the transition state (Ts) and the bound state (B) represents an estimate of the ‘activation energies’ for the inhibitor unbinding processes and so it is related to the drug unbinding rate.

### Analysis

The root-mean-squared fluctuation (RMSF), hydrogen bond (H-bond) and cluster analysis at the equilibrium regions of the TG and CG system have been performed using the Gromacs tools. The analysis have been separately performed for the conformations close to each local minimum, defined as the locus of CVs for which a grid search minimization path ended in the local minimum itself. The total trajectories have been divided in subtrajectories depending on the region (in the CVs subspace) on which each frame was positioned. The conformations with an energy difference from the corresponding local minimum <20 kJ/mole have been assigned to the minimum and used for RMSF, H-bond and cluster analysis. The RMSD-based clusterization of the inhibitor conformations has been carried out with the gromos method (38) implemented in the g_cluster gromacs tool, using a cutoff of 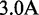.

The fluctuations of DNA bases for each subtrajectory have been calculated using the g_rmsf gromacs tool. The RMSF is defined as follows:

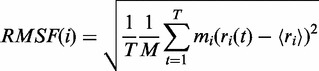

where T is the total number of frames used for analysis, *m_i_* is the mass of i-th atom, 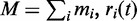 is the position at time *t* of the i-th atom and 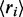 is the average position of the i-th atom in the frames used for analysis.

The H-bond analysis of the various minimum states has been carried out with the g_hbond gromacs tool, using the default cutoff parameters 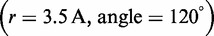. The hydrogen bonds percentages of existence have been calculated from g_hbond output with an in-house written python script.

**Figure 1.**
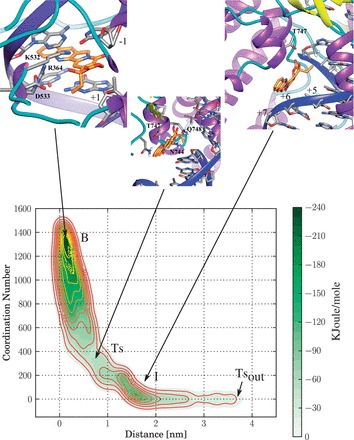
Iso-Energetic Contour plot of FES of CPT unbinding from the TopIB–DNA binary complex having a ‘TG’ cleavage site.

**Figure 2.**
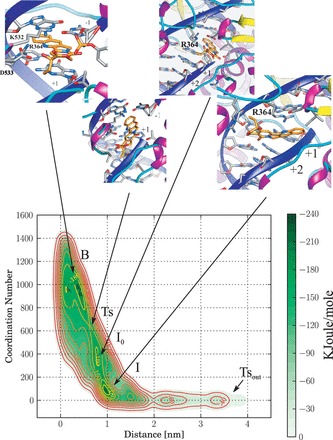
Iso-Energetic Contour plot of FES of CPT unbinding from the TopIB–DNA binary complex having a ‘CG’ cleavage site.

**Figure 3.**
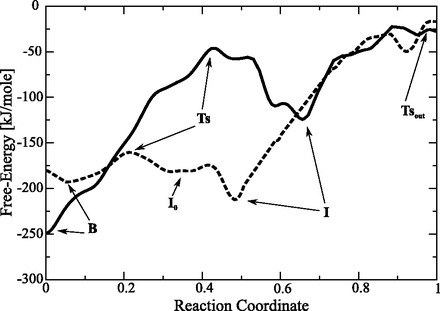
Free-energy profile of CPT unbinding from the TopIB–DNA binary complex, calculated along a minimum energy path, as a function of the generalized ‘Reaction Coordinate’ (0.0 = ‘B’ state, 1.0 = final step of metadynamics). Full line: TG binding site system. Dashed line: CG binding site system.

**Figure 4.**
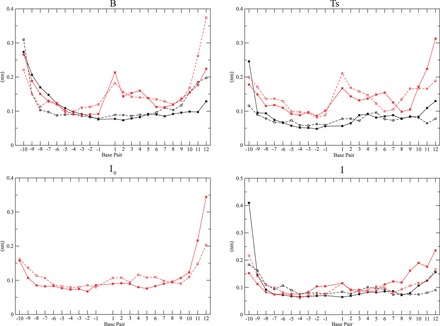
Per residue RMSF of DNA bases calculated in the B, in the Ts in the ‘transient’ (only for the CG-system) and in the ‘stable’ Intermediate state (*I*_0_ and I, respectively). Black line: TG binding site system. Gray lines: CG binding site system. Full line: Nonscissile strand. Dashed line: Scissile strand.

Contours in Figures 1 and 2 have been generated with Matplotlib (v.1.0.0), plot in Figures 3 and 4 have been made with Grace (v5.1.23), the structures represented in insets of Figures 1 and 2 have been drawn with UCSF-Chimera (v1.8). All the Figures have been assembled using the open-source vectorial graphic software Inkscape (v0.48).

## RESULTS

### CPT unbinding from the TG cleavage site

The FES corresponding to the CPT unbinding from the TopIB–DNA complex containing the TG cleavage site, plotted as a function of CPT coordination number and distance from the center of mass of the base pairs flanking the drug, is reported in Figure 1. The surface shows the presence of two deep minima corresponding to the B and the intermediate state (I) separated by a Ts (Figure 1). Finally, a transition state (Ts_out_) at 4-nm distance (Figure 1) identifies the final step of complete unbinding of the CPT from the TopIB–DNA binary complex. The B state is characterized by a large number of contacts and a distance between the CPT and the binding site close to zero, while the I state has a low number of contacts and a distance of CPT from the binding site of ∼1.6 nm (Figure 1). The coordinates corresponding to the conformations belonging to the three states identified in the FES have been extracted and clusterized. The representative structures of the most populated cluster of the B, Ts and I states are reported in the insets of Figure 1. In the B state CPT is intercalated at the cleavage site, in the Ts state it is only partially contacting the cleavage site and in the I state CPT is no longer stacking with the DNA bases. The inter-residues, the residues–DNA in proximity of the cleavage site and the CPT–residues H-bonds, present in >25% of conformations in at least one of the equilibrium states are reported in Table 1.

**Table 1.** Percentage of existence of relevant hydrogen bonds observed for CPT simulation in the TG system, in the B state, in the Ts state or in the I state

Donor	Acceptor	B (7512)	Ts (22)	I (631)
Lys532	CPT	49	0	0
Asn745	CPT	0	27	0
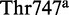	CPT	0	5	30
CPT	Thr747	0	0	36
Gln748	CPT	0	68	0
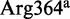	A 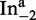	96	96	91
Arg364	A 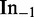	99	100	96
Lys532	A 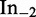	4	46	7
Lys532	T 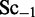	51	9	36
Asn722	T 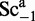	86	91	85
Thr718	G 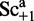	48	0	8
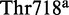	G 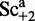	99	0	99.7
Arg364	Asp533	99.9	100	99.8

In the header, the number of frames used for the analysis is reported in parentheses.

In*_n_*: n-th position of intact strand. Sc*_n_*: n-th position of scissile strand.

^a^Backbone.

In the B state only one stable interaction between the CPT lactone ring and the Lys532 residue of TopIB (49%) is observed, while several H-bonds between the TopIB residues and the DNA bases near the TG cleavage site are detected (Table 1). In detail, the Arg364 lateral chain forms an extremely stable (99%) H-bond with the adenine in position −1 of the intact strand complementary to the thymine in position −1 of the TG cleavage site. A stable (96%) aspecific interaction between the backbone of Arg364 and the phosphate of the adenine in position −2 of the intact strand is also observed. Lys532 forms a relatively less stable H-bond (51%) with the thymine in position −1 of the scissile strand, Asn722, the residue next to the catalytic phosphotyrosine 723, a stable interaction (86%) with the phosphate of thymine in position −1 and Thr718 two H-bonds (48 and 99%) one with the backbone of the guanine base in position +1 and another one with the guanine in position +2 of the scissile strand. Arg364 also interacts with the Asp533 residue forming a stable salt bridge. This pattern of interaction is similar to what was observed in the previously reported classical MD simulation of the TPT ternary complex (21).

In the Ts state (inset Figure 1), CPT is shifted toward the DNA major groove side. In this state the lactone group of the drug loses its H-bond interaction with Lys532 observed in the B state and forms two H-bonds with the lateral chain of Asn745 and Gln748 (Table 1). However, several interactions observed in the B state are still present in the Ts state. In detail, the stable interactions of Arg364 with the DNA base in position −1 and −2 are maintained in this conformation. Lys532 temporarily switches its interaction from the base in position −1 of the scissile strand to the base in position −2 of the intact strand, while the interaction between Asn722 and thymine in position −1 is still present in the Ts state (Table 1). The main difference is observed at the level of Thr718 that completely loses the H-bond with the backbone of the DNA bases in position +2 and +1 of the scissile strand. Arg364 maintains an extremely stable salt bridge interaction with Asp533.

Following CPT unbinding, the drug reaches the I state. In this stable configuration, the pattern of interaction between TopIB and DNA is slightly modified when compared with the B state. In detail, the salt bridge between Arg364 and Asp533 is fully maintained. The H-bonds between Arg364 and the adenine in position −1 and between the Arg364 backbone and the phosphate of the adenine in position −2, both in the intact strand, are also maintained. The H-bond between Asn722 and the thymine in position −1 of the scissile strand is also present (Table 1). The percentage of interaction between Lys532 and thymine in position −1 slightly decreases from 51 to 36%. The interaction between Thr718 and the phosphate group of guanine in position +1 partially reappears and the interaction between Thr718 and the DNA phosphate in position +2, both in the scissile stand, is fully formed, returning to the values observed in the B state. In the I state the CPT has of course a different position and its lactone ring forms two H-bonds with the lateral chain of Thr747 located in the helix 23 of the C-term domain, at the interface between the DNA intact strand and the ‘hinge’ helix (residues 432–467) of TopIB, proxymal to the major-groove side of DNA.

From the simulation data the process of CPT unbinding from the TG cleavage site can be described as a sliding of the drug toward the major groove. Such a sliding is associated with an enlargement of the DNA nick width, as measured by the distance between the centers of mass of the bases in position +1 and the bases in position −1. In fact in the B state the measured average distance is 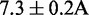, in the Ts state rises to 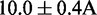, while in the I state the value lowers to 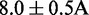 (Table 2). The transient enlargement at the level of the cleavage site in the Ts state is owing to the large volume of the CPT bulky ethyl group that, during the sliding, must cross the DNA helix axis and is coupled with the loss of the H-bond interaction between Thr718 and the DNA bases in position +1 and +2 (cfr. Figure 4, Tables 1 and 3).

**Table 2.** Average distance between the bases in position −1 and +1 in the equilibrium states of the FES of CPT unbinding from the TG and the CG system

Equilibrium State	TG system	CG system
B state	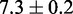	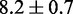
Ts	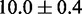	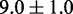
*I*_0_ state	n.a.	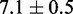
I state	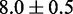	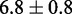

Distances are reported in 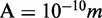.

n.a., not applicable.

**Table 3.** Percentage of existence of relevant hydrogen bonds observed for CPT simulation in the CG system, in the B state, in the Ts state, in the *I*_0_ state or in the I state

Donor	Acceptor	B (1932)	Ts (151)	*I*_0_ (1185)	I (749)
Arg364	CPT	59	24	0	0
Thr718	CPT	61	58	1	0
CPT	Asp533	33	27	1	0
CPT	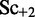	0	0	52	6
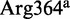	A 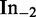	94	97	83	63
Arg364	G 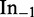	34	57	70	5
Lys532	A 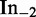	47	42	32	11
Lys532	C 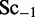	20	22	21	44
Asn722	C 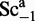	98	99	99	99
Asn722	G 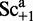	1	0	43	69
Thr718 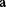	G 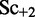	0.2	0	35	90
Arg364	Asp533	60	72	99.8	99.9

In the header, the number of frames used for the analysis is reported in parentheses.

In*_n_*: n-th position of intact strand. Sc*_n_*: n-th position of scissile strand.

^a^Backbone.

### CPT unbinding from the CG cleavage site

The FES corresponding to the CPT unbinding from the CG cleavage site is reported in Figure 2. The surfaces show some remarkable differences when compared with the TG system; in fact, in this case the Ts state separates the B state from two intermediate states *I*_0_ and I, also in this case a transition state Ts_out_ is identified as the final step of the unbinding. Another interesting difference is that the B and Ts states are separated by a relatively small activation energy as can be better appreciated plotting the energy profile, along a minimum energy path, as a function of a generalized reaction coordinate for both the TG and the CG system (Figure 3). Moreover the B state of the CG system is characterized by a lower number of contacts between CPT and DNA when compared with the TG system and by the presence of three substates (labeled 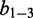 in Figure 2), whose existence can be related to the low energy of the Ts state. As a matter of fact, the low energy difference between both the B and Ts and the Ts and *I*_0_ states, permits CPT to easily cross the Ts, switching from B to *I*_0_ and from *I*_0_ to B. In the *I*_0_ state the drug can undergo a 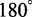 rotation and from this flipped conformation can return into the binding site in a less stable conformation, reaching several sub-B states.

The CPT inter-residues, the residues–DNA in proximity of the cleavage site and the CPT–residues H-bonds present in >25% of conformations in at least one of the equilibrium states of the CG system are reported in Table 3. In the B state CPT interacts with Arg364 and Thr718 through two stable H-bonds (59 and 61%, respectively) and with Asp533 in the 33% of the frames. The Arg364 residue forms a strong (94%) H-bond through its backbone with the adenine in position −2 of the intact strand and a less stable interaction (34%) through its lateral chain with the guanine in position −1. Lys532 interacts, with the adenine in position −2 of the intact strand (47%) and for a low percentage of frames (20%) with the cytosine in position −1 of the scissile strand. Asn722 forms a single stable H-bond (98%) with the phosphate of the cytosine in position −1 of the scissile strand. Arg364 forms a relatively stable salt bridge with Asp533 (60%).

In the Ts state (see the inset of Figure 2 for a representative structure) CPT is shifted toward the DNA minor groove but the H-bond interactions of the drug with Arg364, Thr718 and Asp533 of Top1B are maintained, although slightly decreased in percentage of existence, being 24, 58 and 27%, respectively (Table 3). The H-bonds between DNA and the aminoacidic residues in proximity of the cleavage site are also maintained in the Ts state when compared with the B state, with a slight increase in the percentage of existence (Table 3).

In the *I*_0_ state, CPT is in proximity of the DNA minor groove downstream the cleavage site, and all the H-bonds observed in the B state and in the Ts state are lost. A new H-bond is found between the keto-oxygen on the D-ring of CPT and the amino group of the guanine of the scissile strand in position +2. This interaction occurs because of a rotation of CPT around the axis parallel to its aromatic plane. In the *I*_0_ state the H-bond interaction of Arg364 and Lys532 with the DNA at level of the cleavage site are maintained (Table 3). Asn722 keeps forming a stable interaction with the phosphate group of cytosine in position −1 of the scissile strand, and forms a new interaction through its backbone with the terminal O5′ of the guanine in position +1, an interaction that is never observed in the TG system. A new interaction also occurs between Thr718 and the guanine in position +2, not present in the B and in the Ts state. The salt bridge between Arg364 and Asp533 increases its stability, passing from 60 to nearly 100%.

In the I state, the CPT is not anymore contacting DNA or the protein through stable H-bonds, but it is still in a pocket formed by the DNA minor groove, the loop preceding the linker-domain (residues 634–640) and the loop enclosing Arg364 (see inset in Figure 2). In this state, the interaction between Arg364 and the bases in position −1 and −2 decreases (Table 3). In the case of Lys532, the percentage of the H-bond with the adenine in position −2 decreases, while the one with cytosine in position −1 increases (Table 3). The H-bond of Asn722 with the same cytosine in position −1 is fully maintained and the interaction with the guanine terminal O5′ increases up to 69%. The interaction between the Thr718 backbone and the DNA base in position +2 becomes stable (90%) and the salt bridge between Arg364 and Asp533 maintains its full stability (Table 3).

Analysis of the distance of the center of mass of the bases in position +1 and −1 of the cleavage site of the CG system indicates a value of 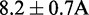 in the B state, of 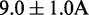 in the Ts state and of 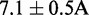 and 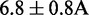 in the *I*_0_ and in the I state, respectively (Table 2). The lower enlargement of the two bases at the level of the Ts state when compared with the TG system is correlated to the different way of escape followed by CPT in the two systems, as it can be appreciated from the trajectories of the CPT unbinding from the TG depicted in Supplementary Figure S1. CPT escapes from the binding pocket of the TG cleavage site toward the TopIB N-terminal side, while in the CG system the direction of unbinding is occurring from the side of the linker domain. In the latter case, the direction of CPT unbinding permits the drug to leave the cleavage site without the need to cross the DNA helix axis and so without inducing the large interbases enlargement observed in the Ts state of the CG system (Table 2).

### DNA flexibility

The DNA flexibility has been analysed in terms of RMSF for all the equilibrium states of the TG and CG system. In the B state, the DNA bases from position −1 to position +6 are significantly more flexible in the CG than in the TG system (Figure 4A). In detail, the base pairs in position +1 reach a fluctuation of 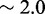 Å in the CG system, and of 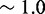 Å in the TG system. This large difference is still observed in the Ts state (Figure 4B), while in the *I*_0_ and in the I state the RMSF value of the CG system decreases reaching values comparable with the ones observed in the TG systems. The high DNA flexibility is linked with the lower interaction of the protein with the DNA cleavage site in the CG system when compared with the TG system and in particular to the different percentage of interaction of Thr718 with the bases in position +1 and +2 of the scissile strand in the B state (Tables 1 and 3). This is confirmed by the observation that the downstream DNA RMSF decrease, when passing from the B to the *I*_0_ state in the CG system, coincides with the restoring of the H-bond between Thr718 and the G+2 base (Table 3). Indeed this interaction is present in both the B and I state of the TG system, but only in the *I*_0_ and I state in the CG system (Tables 1 and 3).

### IQN unbinding from TG and CG cleavage site

The same simulative approach has been applied to the TopoIB–DNA–IQN ternary complex because IQN and its derivatives are known to not display the same sequence specificity of CPT but to be able to stabilize TopoIB-mediated cleavage at both TG and CG sites (7,8). The energetic profile of IQN unbinding from both the TG and the CG system are similar and show comparable differences between the B and the Ts (Supplementary Figures S2 and S3). Moreover, in both systems, IQN follows an unbinding path on the side opposite to the linker domain (data not shown), as observed for CPT in the TG system.

The hydrogen bond analysis (Supplementary Tables S1 and S2) shows that the network between IQN, TopIB and DNA is similar in the two systems. In the B state of the TG and CG system, Arg364 forms stable Hydrogen Bond (HB) with the highly polar keto-oxygen of the IQN indeno-moiety, with the DNA bases in position −1 and −2 of the intact strand and a salt bridge with Asp533. Lys532 interacts with the base in position −1 of the scissile strand in the TG and in the CG system (respectively in 49 and 38% of the frames).

Following the unbinding path, IQN slides toward the DNA major groove, and in the Ts state all the interactions between TopoIB and DNA and in particular the ones involving Arg364, observed in the B state, are maintained. The only exceptions concern the HB between Lys532 and the DNA base in position −1 that is present only in the CG system and shows an increase when compared with the B state (from 38 to 67%) and between Thr747 and IQN that is observed only in the TG state (15%).

Differences are observed at the level of the I state where in the TG system IQN forms four HB with Asn745, Thr747, Gln787 and Asn430 (16, 9, 9 and 43%, respectively), while in the CG system IQN forms a single HB with Gln200. Notwithstanding the differences in the IQN-TopIB HB network, the drug is found in the same region: at the interface between the ‘hinge’ helix (res 432–467), the C-ter domain (res 713–765) and the N-ter (res 1–214), downstream of the DNA cleavage site. The interactions between TopoIB and DNA, observed in the previous states, and in particular the ones involving Arg364 are maintained in both systems, and the interaction between Lys532 and the base in position −1 reappears also in the TG system although with a percentage (16%) lower than in the CG system (56%).

Analysis of the distance between the center of mass of base pairs in position +1 and −1 (Supplementary Table S3) indicates that during IQN unbinding there is not any significant enlargement of the cleavage site in both TG and CG system, likely due to the absence, on the molecular scaffold of IQN, of the rigid and bulky E-ring found on CPT.

## DISCUSSION

The process of CPT unbinding from the TG and the CG cleavage site displays interesting differences when investigated via a metadynamics approach. The first asymmetry observed between the two systems concerns the energy difference between the B and the Ts states that is much lower in the CG system than in the TG system (Figure 3) and it is likely correlated with the different trajectories of CPT unbinding observed in the two systems (Supplementary Figure S1). Following the path of unbinding, in the I state visited in the simulation of the TG system, CPT is located in the pocket at the interface between the protein and the DNA major groove toward the N-Terminal domain of TopIB, while in the I state of the CG system CPT is located in proximity of the linker domain between the protein and the DNA minor groove (inset of Figures 1 and 2, respectively). It is amazing that the large free-energy profile variation between the two systems, resulting in a different CPT unbinding trajectory, is dictated by the presence of a single different couple of bases namely a cytosine in place of a thymine in position −1 of the scissile strand with the consequent presence of a guanine in place of an adenine in position −1 of the intact strand. Previous works on isolated drug–bases systems have proposed that the apolar nonbonding CPT–DNA interaction has a role in determining the specificity (9–14). However, the results here presented indicates that the observed differences are not simply ascribable to a different stacking of the drug with the mutated bases, but also to the protein and to the different network of noncovalent protein–DNA and protein–CPT interactions occurring in the two systems.

The H-bond analysis shows that the pattern of interaction between TopIB and DNA is different in the two systems. In the B state, Arg364 and Lys532 interact with the DNA bases in position −1 more firmly in the TG than in the CG system (Tables 1 and 3). At the same the time the salt bridge interaction between Arg364 and Asp533 is more stable in the TG system than in the CG system. Arg364 plays an important role in the interaction of TopIB with the cleavage site (15–17,20,21,39) and the loosening of its H-bond with the guanine in position −1 and of the Arg364–Asp533 salt bridge permits, in the CG system, the direct interaction of these two residues with CPT (Table 3). The rearrangement of the pattern of interaction of Arg364 and Asp533 also perturbs Lys532, disrupting its H-bond with the drug in the CG system, while this is the only stable interaction experienced by CPT in the B state of the TG system (Table 1). In this latter system, the presence of the Lys532-CPT H-bond does not permit the drug to follow the way of escaping toward the DNA minor groove, forcing it to slide toward the major groove side. On the other hand, the presence in the CG system of the double Arg364 and Asp533-CPT interaction (Table 3) and the different orientation of Lys532 permits a slight rotation of the drug, giving the possibility to CPT to leave the cleavage site toward the DNA minor groove, favoring its interaction with Thr718. In line with these observations, in the TG system, Thr718 shows a stable interaction with the DNA scissile strand in position +1 and +2 (Table 1), while in the CG system, this interaction is observed only after the drug displacement into the *I*_0_ and I state (Table 3). The different level of interaction of Thr718 with DNA is also correlated with the larger fluctuation of DNA bases in position +1 and +2 of the CG system when compared with the TG one (Figure 4), confirming the important role of Thr718 in defining the correct orientation of the free terminal O5′ to permit the DNA religation (40).

The results of the metadynamics simulations here presented provide an atomistic explanation of the experimental evidence of a strict need for a thymine in position −1 for the CPT-mediated stabilization of the TopIB–DNA covalent complex. Experimental analysis of the frequency of the bases at the cleavage site has in fact unambiguously demonstrated that CPT can stabilize the covalent TopIB–DNA complex only when a thymine is present in position −1 (6). Our work shows that when a thymine is present in position −1 of the TopIB cleavage site (TG system) the H-bond network between the protein and DNA forces CPT to leave its binding site crossing the DNA helix, an event that implies an high energetic cost, requiring the enlargement of bases at the cleavage site to permit the passage of the ethyl group on the CPT E-ring. When a cytosine is present in position −1 (CG system) the different H-bond network permits CPT to leave the cleavege site without crossing the DNA helix axis.

Moreover the less rigid H-bond network around the cleavage site confers a relatively large flexibility to the scissile strand as confirmed by the RMSF values (Figure 4) and by the relatively large interbases distance in the B state of the CG system (Table 2) lowering the energetic cost for the escape of CPT from its binding site. This result demonstrates that besides the stacking interaction between the drug and the bases at the cleavage site, on which previous simulative studies on isolated base–drug system have focused (9–14), an important role in determining the different stabilization of TopIB–DNA–CPT ternary complex is played by the protein and by the different H-bond network occurring in the TG and CG system. It is interesting to notice that a direct role of the protein in mediating the sequence specificity of the cleavage sites stabilization by inhibitors has been also suggested in the case of topoisomerase II (41).

On the other hand, the unbinding process of Indenoisoquinole, a TopIB inhibitor that can stabilize the protein both on the TG and CG cleavage sites, shows a similar free-energy profile in both the TG and CG system. Consistently, the HB network between TopoIB, DNA and the drug is similar in the two systems, likely owing to the presence on IQN of the highly polar keto-oxygen that, when the drug is intercalated between the DNA bases, protrudes into the DNA minor groove, permitting the formation of an extremely stable HB between the drug and Arg364 in both the TG and the CG systems, without any significant perturbation of the Arg634 interaction with DNA and Asp533, avoiding the modification of the TopoIB–DNA interaction pattern that induced the CPT drug to follow a different unbinding path in the CG system.

## CONCLUSION

Our simulation provides a two-step model for the CPT unbinding process. In the first step, the drug passes from the B state, where the drug is stacked at the cleavage site, to the I state, where the interaction with the DNA is not anymore present; in the second step, CPT exits from the I state reaching the solvent. The free-energy barrier between the B and the Ts state (Figures 1–3) is much lower in the CG than in the TG system, not permitting the stabilization of the drug in the B state of the CG system and letting it to easily reach the I state and finally the Ts_out_ state. According to the Ts theory, the height of the energy barrier is directly related to the rate of unbinding, so the high energy difference between the B and the Ts states, observed in the TG system, is indicative of a low CPT unbinding rate, while the lower energy, observed in the CG system, is consistent with a high unbinding rate. These observations, coupled with the analysis of TopoIB–DNA H-bonds, provide an explanation for the experimental evidence of the need for a thymine in position −1 of the drug-stabilized cleavage complex (6). The interaction of Arg364 and Lys532 with the nucleobases in position −1 are proposed to play a major role in determining the sequence specificity, imposing a different unbinding path to CPT. A role of Thr718 and the fluctuation of the DNA bases in position +1 and +2 is also observed, suggesting the importance of the protein–DNA interplay in defining the sequence specificity of the CPT drug at the stabilized cleavage site (6). The occurrence of a different unbinding path depending on the cleavage site sequence can be even enhanced ‘*in vivo*’ owing to the presence of the N-terminal domain that can sharpen the preference for the TG cleavage site. The N-term region can represent, in fact, an additional obstacle for the CPT unbinding that is not felt in the alternative unbinding path observed in the CG system.

The results for the IQN suggest that the ability of these classes of compounds to stabilize TopIB-mediated cleavage site at both CG and TG sites is related to the presence of the polar keto-oxygen that permits a stable HB interaction with the protein bypassing the modification of TopoIB–DNA HB network, observed in the case of CPT. The ability of the here presented metadynamics simulative setup to qualitatively reproduce and explain at atomistic level experimental results provide evidence of the power of the method that can be extended to the analysis of other TopoIB inhibitors.

## SUPPLEMENTARY DATA

Supplementary Data are available at NAR Online.

## FUNDING

The computational time was obtained through the CASPUR (now CINECA) Standard Grant [std12-013] ‘Meta-Dynamics simulation of human topoisomerase IB inhibitors ternary complexes’ (to A.C.). This work was partially funded by the AIRC project [10121 to A.D.]. Funding for open access charge: Italian Association for Cancer Research (AIRC) [10121].

*Conflict of interest statement.* None declared.
